# Research on Employee Sense of Gain: The Development of Scale and Influence Mechanism

**DOI:** 10.3389/fpsyg.2020.568609

**Published:** 2020-10-02

**Authors:** Yinhua Gu, Yingyao Yang, Jing Wang

**Affiliations:** ^1^School of Management Science, Chengdu University of Technology, Chengdu, China; ^2^School of Management, Shanghai University, Shanghai, China

**Keywords:** employee sense of gain, scale development, influence mechanism, organizational support theory, supportive human resource practices

## Abstract

Employee satisfaction is a main source of firms’ competitive advantages. Employee sense of gain (ESG) is defined as the subjective feeling of getting various objective benefits due to employees’ efforts at work. It appropriately reflects employee satisfaction with the objective needs and their subjective perception of the firms, which affects their attitude, behavior, and work performance. Although ESG is gaining increasing interest in human resource research and managerial practice, literature on its conception, measurement, and contribution to human resource theory is still limited. Study 1 developed an integrative framework of concept of ESG and reported the development and validation of a scale measuring ESG. Based on the exploratory factor analysis of 201 valid questionnaires responded by enterprise staffs, the initial scale was formed. Through confirmatory factor analysis of 172 questionnaires, the formal scale was obtained. The multiphase scale development process resulted in a 14-item ESG scale measuring two dimensions: employee’s material sense of gain and employee’s spiritual sense of gain. Study 2 investigated the influence mechanism of ESG based on statistical analysis of 254 valid questionnaires. The results showed that ESG was influenced by all three dimensions of supportive human recourse practices (SHRP), whereby the influences were mediated by perceived insider status (PIS). Results also suggested that leader political skill (LPS) moderated the effect between SHRP (fairness of rewards and punishments, growth opportunity) and PIS positively. Overall, this research provided a reliable and valid measurement scale of ESG in the Chinese setting and explored the influence mechanism, as well as boundary conditions. Managerial implications were provided from the perspectives of organizations, leaders, and employees.

## Introduction

In the increasingly fierce war for talents, showing care to employees rather than merely paying attention to their professional performance is one of the most important means for organizations to win competitive edges. With the continuous development of society, the status of employees in the organization is continuously rising. A globally identified concept has been formed that the organization not only values the contribution of employees but also cares for their welfare (Eisenberger et al., 1986; Allen et al., 2003). Sense of gain, in Chinese society concept’s nature setting, aims to describe the living conditions of citizens. It refers to people’s subjective evaluation of their benefit in the process of social development (Cao and Li, 2017). Employee sense of gain (ESG) reflects employees’ subjective feelings of getting various benefits from their efforts at work, which pertinently suggests their satisfaction and their perception of the firm. Due to the significant effects on employees’ attitude, behavior, and work performance, ESG is increasingly becoming an important theme in human resource theory and practices.

Despite various efforts aimed at conceptualizing and operationalizing of ESG, questions regarding its conception and measurement as well as the influence mechanism and boundary conditions remain. The concept and definition of ESG not yet determined, different scholars have different understandings for the concept and connotation of it. It is generally believed that the sense of gain is a subjective feeling based on objective gain (Ding, 2016). ESG is a multilevel demand, which is mainly manifesting in the material level of the improvement of the material standard of living and the spiritual level of self-value achievement (Zheng and Chen, 2017). At present, most relevant researches studying ESG are proposed on macro perspectives of political science or sociology. However, ESG is a subjective feeling that requires micro-level studies. ESG is the application of sense of gain in the organizational context, which more closely reflects the extent of employees’ satisfaction of needs objectively and their recognition of the organization subjectively (Jiang and Zhang, 2016). However, relevant theoretical research related to ESG is lacking, the concept of ESG has not yet been clearly defined, the influence mechanism of ESG has not been revealed, and even measurement tools are deficient. These have caused a huge obstacle to the further exploration of ESG.

Against this background, this article seeks to shed light on the nature of sense of gain, its measurement, and its relation to other theoretically and managerially meaningful constructs. To these purposes, this research provides a reliable and valid measurement scale of ESG in Chinese setting and explores the influence mechanism, as well as boundary conditions. Specifically, study 1 was conducted to determine the definition of ESG and develop measurement scale. Firstly, the concept of ESG was sorted out and defined, and the initial scale was developed by literature research and expert discussion. Next, exploratory factor analysis, confirmatory factor analysis, and reliability test were conducted on the basis of the initial scale to form the formal scale. Study 2 aims to explore the antecedents, the influence mechanism, and the boundary conditions of ESG based on the organizational support theory.

## Study 1: The Scale Development of Employee Sense of Gain

### Methods

In this study, a qualitative and quantitative method was adopted to develop a scale of ESG (Hinkin, 1998). Firstly, according to the theoretical boundary of the concept, the coverage of the ESG measurement indicators was determined and the initial items were developed accordingly. Next, human resource management experts, entrepreneurs, and doctoral students were invited to discuss and optimize the initial items. Later, a small-scale trial was run to adjust the expressions that were hard to comprehend or were ambiguous. Afterward, the first batch of questionnaires was distributed for the prediction test, and exploratory factor analysis and reliability test were conducted on the sample data to tentatively form the scale of ESG. Finally, the second batch of data was collected to conduct confirmatory factor analysis on the initial scale of ESG, so as to form the formal scale.

### Procedure

#### Definition of Employee Sense of Gain

In February 2015, at the Chinese 10th meeting of the central leading group for comprehensive deepening, president Xi Jinping of China put forward the concept of “sense of gain” for the first time. In recent years, it has been a heated topic in the academic field. Sense of gain is a newly localized concept proposed on the basis of the Chinese social context to describe the living conditions of citizens (Jiang and Zhang, 2016). Sense of Gain is a subjective feeling formed on the premise of both participation and contribution and the basis of objective gain (Ding, 2016; Tang, 2017). It indicates an integration of objective acquisition and subjective feeling (Ma and Liu, 2017). Objective gain not only is limited to interests in material and economic aspects but also includes the right to know; the right to participate, to express, and to supervise; and the opportunity for self-actualization (Cao and Li, 2017). As a kind of subjective feeling, different individuals may have various perceptions for the same objective acquisition. Sense of gain is a subjective evaluation of one’s benefits and a personal consciousness of internal satisfaction and pleasure (Tang, 2017). In addition, sense of gain also reflects multiple levels of demands (Zhang, 2016), not only on the material dimension (such as the improvement of material living standard, the demand for economic benefits) but also on the spiritual dimension (such as the fair pursuit for dreams, the dignity of life, the realization of self-worth).

Employee sense of gain is the application of sense of gain in the organizational context. It reflects the essential characteristics of “sense of gain” and shows its particularity in the organizational context. Currently, there is no acknowledged definition of ESG in the academic community. Based on relevant literature, this study defined ESG as the subjective feeling of getting various objective benefits due to employees’ efforts at work. This definition is embedded with three implications: efforts at work serve as the precondition for ESG; various objective benefits are the basis of ESG; and subjective feelings are viewed as the kernel of ESG.

#### Indicators of Employee Sense of Gain

In March 2018, the Chinese National School of Development at Peking University, Chinese Academy of Social Sciences, together with other institutions published the Better Life Index, which included 28 indicators that are most closely related to sense of gain. These indicators were extracted by scientific methods after a large number of interviews. Ranging the correlation degree from high to low, the 28 indicators are as follows: mentality and emotion, health condition, income level, domestic harmony, power of example, ecological environment, awareness of law, cultural confidence, leisure-time life, self-worth, price level, social security, education and training, housing condition, interpersonal relations, benefit level, convenience for consuming, quality of old-age security, social identification, promotion opportunity, development of next generation, spiritual pursuit, salary level, group culture, working strength, government’s consciousness of service, and the relationships between colleagues and working efficiency of government.

This study would determine the indicators for ESG based on the 28 indicators above. The expert group approach was adopted to examine these indicators through the perspective of organizational context and determined whether they should be kept, transformed, or deleted. First, retained indicators that can be directly incorporated into the organizational context, including “mentality and emotion,” “health condition,” “power of example,” “leisure-time life,” “self-worth,” “promotion opportunity,” “benefit level,” “salary level,” “work strength,” and “the relationships between colleagues.” Second, transformations could be applied indirectly to indicators in an organizational context, including converting “income level” to “salary level,” “ecological environment” to “working environment,” the “awareness of law” to “rules and regulations,” “social security” to “medical security,” “education and training” to “staff training,” “housing condition” to “housing security,” “interpersonal relations” into “the relationship between colleagues,” “quality of old-age security” to “old-age security,” “social identification” to “organizational identification,” “spiritual pursuit” to “self-worth,” “domestic harmony” and “development of next generation” to “work-family enrichment,” “cultural confidence” and “group culture” to “organizational culture,” and “government’s consciousness of service,” and “working efficiency of government” to “management service.” Third is the eliminated indicators that could not be included in the organizational context. “Price level” and “convenience for consuming” were deleted. Finally, an initial scale for ESG composed of 20 indicators was formed (see Table 1).

**TABLE 1:** The initial scale of ESG.

Serial number	Indicator	Sources of indicators (sense of gain)	Item	Category
ESG101	Health condition	Health condition	I can keep in good health condition in the company.	ESG1
ESG102	Salary level	Salary level, income level	I am satisfied with the salary level in the company.	
ESG103	Benefit level	Benefit level	I am satisfied with the benefit level in the company.	
ESG104	Old-age security	Quality of old-age security	I am satisfied with the old-age security provided by this company.	
ESG105	Housing security	Housing condition	I am satisfied with the housing security provided by this company.	
ESG106	Medical security	Social security	I am satisfied with the medical security provided by this company.	
ESG107	Working environment	Ecological environment	I am satisfied with the working environment in the company.	
ESG108	Working strength	Work strength	I am satisfied with the working strength in the company.	
ESG109	Staff training	Education and training	I am satisfied with the staff training provided by the company.	
ESG110	Promotion opportunity	Promotion opportunity	I am satisfied with the promotion opportunities offered by this company.	
ESG201	Organizational identification	Social identification	When others evaluate the company, I feel like I’m evaluating myself.	ESG2
ESG202	Workplace mentality and emotion	Mentality and emotion	I have a good mood when I work in this company.	
ESG203	Rules and regulations	Awareness of law	The company has sound rules and regulations and implements them strictly.	
ESG204	Organizational culture	Cultural confidence, cultural confidence	I am satisfied with the cultural atmosphere of the company.	
ESG205	The relationships between colleagues	The relationships between colleagues, interpersonal relations	I am satisfied with the relationships between colleagues in this company.	
ESG206	Leisure-time life	Leisure-time life	I am satisfied with the leisure-time life activities organized by the company.	
ESG207	Management service	Government’s consciousness of service, working efficiency of government	I am satisfied with the service consciousness and efficiency of the company.	
ESG208	Self-worth	Self-worth, spiritual pursuit	I can realize my self-worth by working in this company.	
ESG209	Power of example	Power of example	The example of this company can inspire me to make progress.	
ESG210	Work-family enrichment	Domestic harmony, development of next generation	Working in this company helps to promote the harmony of my family.	

#### Initial Scale

First, the indicators of ESG were explained further to form 20 initial items. For example, the item formed by the indicator of “health condition” was expressed as “I can keep in good health condition in the company.” Later on, human resource management experts, entrepreneurs, and doctoral students discussed and reviewed the initial indicators and items and repeatedly revised the expressions. Since ESG is the satisfaction of multilevel demands, this study divided it into two dimensions: employee’s material sense of gain (ESG1) and employee’s spiritual sense of gain (ESG2). ESG1 included sense of gain obtained from income, housing, old age, and medical security. In total, 10 items were designed for ESG1. ESG2 consisted of dream, pursuit, equal rights, self-actualization, and other psychological satisfactions. As a result, 10 items were designed for ESG2. Finally, an initial scale with 20 items concerning both ESG1 and ESG2 is shown in Table 1 after making some minor changes and revisions.

#### Participants

The initial scale was used to issue the first batch of questionnaires to Chinese employees. A total of 240 paper questionnaires were sent out, and 201 of them were valid (the recovery rate was 83.750%). The questionnaire used the Liker five-point scale to evaluate the result from “1” = strongly disagree to “5” = strongly agree. The demographic distribution of valid questionnaires was as follows: first, gender: 135 males (67.2%) and 66 females (32.8%); second, age: 25 years or below (28.9%), 26–30 years (30.8%), 31–35 years (17.9%), 36–40 years (6.5%), 41–45 years (6.5%), and 46 years or above (9.5%); third, education level: high school or below (9.5%), college graduate (32.3%), undergraduate (46.3%), and postgraduate or above (11.9%); fourth, length of service: fewer than 1 year (18.9%), 1–3 years (33.8%), 4–6 years (18.4%), 7–9 years (12.9%), 10–12 years (7.5%), and 13 years or above (8.5%); and fifth, position: senior management or high-level skills (12.4%), middle management or intermediate skill (34.8%), low-level managers or low-level skills (37.3%), and others (15.4%).

### Result

#### Data Analysis

SPSS 20.0 and AMOS 25.0 were applied to analyze the data through exploratory factor analysis, reliability test, and confirmatory factor analysis. Principal component analysis and varimax were adopted to verify the scale.

#### Exploratory Factor Analysis

SPSS 20.0 were implemented to conduct an exploratory factor analysis. To verify whether the data collected by surveys were valid for factor analysis, suitability test and Bartlett’s spherical test were carried out. The KMO was 0.902 > 0.70, the chi-square approximation of Bartlett’s test of sphericity was 2218.987 (*df* = 190, *p* < 0.001), which met the requirements and was valid for exploratory factor analysis on the ESG scale (Kaiser and Rice, 1974).

Principal component analysis and varimax were adopted together. Two factors were extracted with the scree plot. First, the following two items were eliminated by taking 0.400 as the threshold of factor loading (Hinkin, 2005): factor loading on all factors is less than 0.400, and the factor loading on all factors is more than 0.400. Afterward, dimensions with more items should be retained if items from different dimensions coexist in a common factor, while the items with the largest factor loading in other dimensions should be deleted. According to these rules, 6 items were eliminated including ESG201, ESG101, ESG108, ESG107, ESG109, and ESG110 to obtain an ESG scale consisting of two dimensions: ESG1 (factor 2 with 5 items) and ESG2 (factor 1 with 9 items). There were 14 items, and the total variance explained reached 60.758% (see Table 2), which met the standard (Hinkin, 1998).

**TABLE 2:** Exploratory factor analysis results of the scale of ESG.

Serial number	Item	Factor 1	Factor 2
ESG102	I am satisfied with the salary level in the company.	0.298	**0.697**
ESG103	I am satisfied with the benefit level in the company.	0.272	**0.817**
ESG104	I am satisfied with the old-age security provided by this company.	0.122	**0.799**
ESG105	I am satisfied with the housing security provided by this company.	0.164	**0.774**
ESG106	I am satisfied with the medical security provided by this company.	0.291	**0.802**
ESG202	I have a good mood when I work in this company.	**0.709**	0.301
ESG203	The company has sound rules and regulations and implements them strictly.	**0.715**	0.111
ESG204	I am satisfied with the cultural atmosphere of the company.	**0.813**	0.199
ESG205	I am satisfied with the relationships between colleagues in this company.	**0.665**	0.086
ESG206	I am satisfied with the leisure-time life activities organized by the company.	**0.737**	0.185
ESG207	I am satisfied with the service consciousness and efficiency of the company.	**0.708**	0.286
ESG208	I can realize my self-worth by working in this company.	**0.691**	0.240
ESG209	The example of this company can inspire me to make progress.	**0.752**	0.344
ESG210	Working in this company helps to promote the harmony of my family.	**0.682**	0.272
Eigenvalue		6.704	1.802
Variance explained (%)		47.888	12.871

*The factor loadings of the items onto their target factor are in bold.*

#### Reliability Analysis

In order to ensure a high consistency among all items of the scale, this study further tested the reliability by analyzing the Cronbach’s alpha of the scale and taking 0.70 as the standard for the internal consistency reliability (Guielford, 1965). At the same time, if deleting an item was found to be helpful in increasing the reliability coefficient of the scale, the item should be deleted. Each item’s corrected-item total correlation (CITC) should reach 0.500 (Churchill, 1979). The results showed that the scale of ESG1’s Cronbach’s alpha = 0.871 and each item’s CITC was higher than 0.617. The scale of ESG2’s Cronbach’s alpha = 0.905 and each item’s CITC was higher than 0.567. None of them found that the reliability of the subscale could be increased when deleting an item, indicating that the reliability of the ESG scale was relatively high.

#### Confirmatory Factor Analysis

The second batch of questionnaires was distributed to Chinese employees using the 14-item scale of ESG. A total of 200 paper questionnaires were sent out, and 172 of them were valid (the recovery rate was 86.000%). The questionnaire used the Liker five-point scale to evaluate the result from “1” = strongly disagree to “5” = strongly agree. The demographic distribution of valid questionnaires was as follows: first, gender: 112 males (65.1%) and 60 females (34.9%); second, age: 25 years or below (28.5%), 26–30 years (37.2%), 31–35 years (18.0%), 36–40 years (9.9%), 41–45 years (4.1%), and 46 years old or above (2.3%); third, education level: high school or below (9.9%), college graduate (41.3%), undergraduate (39.9%), and postgraduate or above (9.9%); fourth, length of service: fewer than 1 year (26.2%), 1–3 years (33.7%), 4–6 years (17.4%), 7–9 years (14.0%), 10–12 years (6.4%), and 13 years or above (2.3%); fifth, position: senior management or high-level skills (12.2%), middle management or intermediate skills (30.8%), low-level managers or low-level skills (43.6%), and others (13.4%).

**FIGURE 1:**
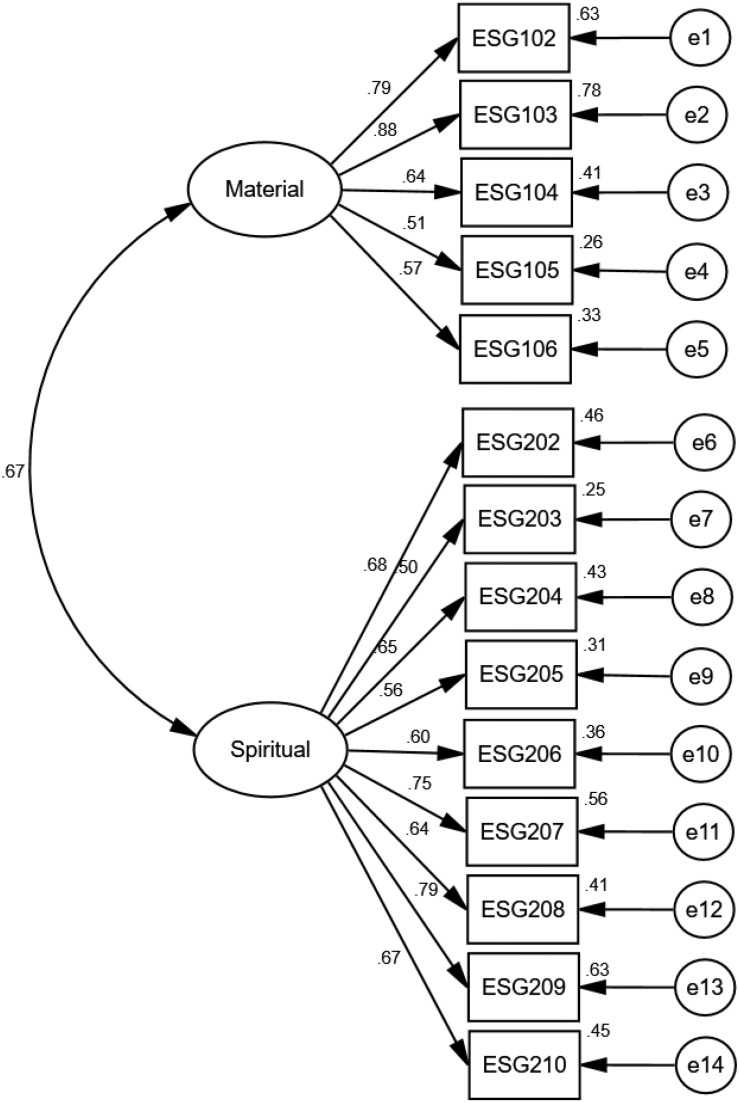
Standardized path diagram for two-dimensional structural equation model of ESG.

AMOS 25.0 was applied to analyze the data collected. By examining 172 valid data, the overall fitting of the model was relatively satisfying. χ^2^/*df* = 1.907, RMSEA = 0.073, RMR = 0.048, GFI = 0.928, AGFI = 0.850, PGFI = 0.645, NFI = 0.860, IFI = 0.928, CFI = 0.927, TLI = 0.912. It was suggested that the fitting optimization index was acceptable and the structure of the model was designed reasonably (Browne and Cudeck, 1992; Byrne, 2001). Therefore, the validity could be further tested by factor loading. The confirmatory factor analysis for factor loading is shown in Figure 1. The result illustrated that the standardized factor loading coefficients of all observable variables on corresponding latent variables were greater than or equal to 0.50, and all of them were verified by the Student’s t test and significant (*p* < 0.001), indicating that the scale had reasonably good convergence.

### Study 1 Discussion

To sum up, study 1 defined the ESG and developed the measurement scale. In this study, ESG was defined as the subjective feeling of getting various objective benefits due to employees’ efforts at work, including both material and spiritual dimensions. ESG1 refers to the sense of material gain in terms of material factors such as salary and benefit level, or old age, housing, and medical security. ESG2 is a sense of gain caused by psychological factors including organizational culture, the relationships between colleagues, leisure-time life, self-worth, and power of example. By applying a combined approach of qualitative and quantitative methods, a scale for ESG consisting of 14 items was developed. In this scale, 5 items were related to ESG1 while 9 were relevant to ESG2, and the total variance explained reached 60.758%.

## Study 2: The Influence Mechanism of Employee Sense of Gain

Flowing study 1, study 2 further explored the antecedents, influence mechanism, and boundary conditions of ESG. According to the organizational support theory (OST), perceived insider status (PIS) as the mediating role, and leader political skill (LPS) as the moderating role, an influence mechanism of supportive human resource practices (SHRP) on ESG was established and tested.

### Theory and Hypothesis

#### Supportive Human Resource Practices and Employee Sense of Gain

Human resource practices are an umbrella term for various policies, means, and systems that affect employees’ behaviors, attitudes, and performance. Different types of human resource practices have various priorities. SHRP are defined as a management practice which an organization invests in and shows recognition on employees’ contributions (Allen et al., 2003). “Support” in this term demonstrates “organizational support” (Meyer and Smith, 2000). High-performance human resource practices emphasize measures such as improving employees’ working capacity, participation in decision-making, and motivation for making individual effort, so as to improve enterprise performance and sustainable competitiveness (Sun et al., 2007). High commitment human resource practices illustrate facilitating organizational goals by strengthening the emotional commitment between the organization and employees (Whitener, 2001). Strategic human resource practices emphasize that human resource management should be highly consistent with corporate strategy (Wright et al., 2005).

From the perspective of employees, SHRP emphasize that the organizations should provide necessary support to employees as well as value their contributions. SHRP mainly provide support from three aspects: fairness of rewards and punishments (SHRP1), participation in decision-making (SHRP2), and growth opportunity (SHRP3) (Allen et al., 2003). SHRP1 refers to the organization’s fair and impartial implementation of rewards and punishments by the procedures so that employees can get fair return for their efforts. SHRP2 is to allow employees to participate in decisions related to their work and to enhance their participation. SHRP3 implies that organizations provide enough training for employees so that they can optimize their abilities and make progress at work. Many scholars have done research on SHRP and discovered that it has positive effects on employees’ behavior such as improving their job satisfaction (Griffeth et al., 2000), organizational commitment (Shore and Wayne, 1993), individual and organizational performance (Datta et al., 2005), and reducing their turnover intention (Huselid, 1995). Therefore, this study infers that ESG is affected by SHRP.

Organizational support theory proposed on the foundation of social exchange theory, and the norm of reciprocity points out that organizations’ support for employees include whether they show concern for employees’ contributions and welfare. OST illustrates that organizations must make commitment employees before employees have commitment to their enterprises (Eisenberger et al., 1986). In this view, the supportive management measures provided by organizations can meet the needs of employees, thus affecting their attitude, behavior, and work performance. Their sense of gain can be improved by more rewards obtained at work.

Organization fairly recognizes and rewards employees, which indicates its concerns for employees’ welfare and willingness to make investment in them. These treatments can affect the employee’s perceived organizational support (Rhoades et al., 2001) and make them recognize that their efforts are fairly repaid, which can improve their sense of gain (Shi, 2017). If organizations encourage employees to play roles in decision-making, it not only can increase their engagement at work but also indicate organizations’ recognition of their contributions. According to Wayne et al. (1997), growth opportunities mark the recognition and emphasis of the organization on employees’ contributions, which also suggests that employees will be given promising support by the organization in the future. These supportive management measures can boost employees’ perception of organizational support, having positive effects on employees and improving their sense of gain. Eisenberger et al. (1990) claim that individuals who receive more support from the organization are more likely to feel obligated to repay the organization (Shore and Wayne, 1993) and improve their performance. Meanwhile, they have expectations for being concerned with and rewarded by their superior so that they may put more efforts into their work (Rhoades and Eisenberger, 2002). SHRP support employees from SHRP1, SHRP2, and SHRP3 and motivate employees to make more efforts so that they can get more rewards and improve their sense of gain. Thus, this study considers that all dimensions of SHRP (SHRP1, SHRP2, and SHRP3) have positive effects on all dimensions of ESG (ESG1 and ESG2). Thus, the following research hypotheses are proposed:

H1a: SHRP1 has positive effects on the ESG1;
H1b: SHRP2 has positive effects on the ESG1;
H1c: SHRP3 has positive effects on the ESG1;
H2a: SHRP1 has positive effects on the ESG2;
H2b: SHRP2 has positive effects on the ESG2;
H2c: SHRP3 has positive effects on the ESG2.

#### Mediating Effect of Perceived Insider Status

PIS proposed by Stamper and Masterson (2002) is defined as employees’ perception of personal space and acceptance as members of an organization and is used to measure employees’ perception of the degree and sense of belonging within the organization (Masterson and Stamper, 2003). Stamper and Masterson (2002) found that perceived organizational support can improve employees’ PIS. According to OST, it can be known that the degree of contribution and concern of an organization to employees determine their attitude toward the organization. The support provided by organizations needs to be perceived by employees to produce perceived organizational support (Eisenberger et al., 1986). Only when employees feel that the organizations respect their interests and value their contributions do they identify themselves as insiders (Amabile et al., 2004).

In a working environment that highlights fairness and justice, it is easier for employees to have benign interaction, mutual support, and cooperation with the organization and its members, thus promoting the formation of PIS (Stamper and Masterson, 2002). The research of Armstrong-Stassen and Schlosser (2011) showed that employees have a higher level of PIS when they think they are treated fairly by supervisors. Employees perceive organizational support from superiors; for example, the decentralization of decision-making power and autonomy by leaders, or employees’ participation in decisions related to their interests to meet the internal needs, has a significant impact on employees’ PIS (Stamper and Masterson, 2002). Generating positive cognitive experience for the support of the organization will enhance employees’ trust in the organization; that is, they will form more emotional identification with the organization (Tsai, 2013) and generate a stronger sense of belonging. The perception of support also encourages employees to consider organizational identification as an important part of their self-identification (Eisenberger et al., 1990). Employees can get more support from the organization, which means that employees have been accepted by the organization and obtain more personal space. The initiative measures taken by the organization to employees through SHRP are regarded as the importance and respect for themselves (Wayne et al., 1997), thus improving employees’ PIS.

Previous studies have found that employees with a higher level of job satisfaction, creativity, organizational commitment, organizational identification, performance, and knowledge sharing have a higher degree of PIS (Stamper and Masterson, 2002; Knapp et al., 2014; Zhao et al., 2014; Guo and Qiu, 2019; Zheng et al., 2019; Guo et al., 2020). PIS, an important psychological perception and cognition degree, conveys a signal to employees that they are important parts of the organization. Employees realize their “internal members” identity in the organization, which improves their sense of “masters” identities in the organization (Wang et al., 2006). The higher the level of PIS is, the more obvious the employees’ working attitudes will be affected, and the lower their turnover intention will be (Knapp et al., 2014). A high level of PIS can also arouse positive emotion toward the organization and promote employees’ intrinsic motivation, thus affecting their performance (Chen and Aryee, 2007). When employees consider themselves parts of the organization, they are more willing to take responsibility and devote themselves (Chen and Aryee, 2007). When employees incorporate the concept of PIS into their self-identification process, their work output will be greatly promoted after certain needs in social emotion are met (Eisenberger et al., 1986; Chen and Aryee, 2007). Therefore, employees with a high level of PIS consider themselves as important members of organizations, thus cultivating positive emotion toward organization, improving their behavior, and enhancing their sense of gain at work.

In conclusion, this study argues that SHRP (SHRP1, SHRP2, and SHRP3) provide organizational support to employees and conveys the signal that they are insiders in organizations. Perceiving that the organization respects their interests and values their contributions has positive influence on the employees’ PIS, generates positive emotion and behaviors, and thus promotes their ESG. Therefore, the following research hypotheses are proposed:

H3a: PIS mediates the relationship between SHRP1 and ESG1;
H3b: PIS mediates the relationship between SHRP2 and ESG1;
H3c: PIS mediates the relationship between SHRP3 and ESG1;
H4a: PIS mediates the relationship between SHRP1 and ESG2;
H4b: PIS mediates the relationship between SHRP2 and ESG2;
H4c: PIS mediates the relationship between SHRP3 and ESG2.

#### Moderating Effect of Leader Political Skill

Political skill is an individual ability to effectively understand others at work and use such knowledge to influence others to act in ways that enhance one’s personal and organizational objectives (Ahearn et al., 2004). Leaders with a high level of LPS tend to have good social acuity, impression management, and interpersonal influence. They can flexibly carry out various interpersonal interactions according to different situations and targets and achieve satisfying goals by different strategies (Harris et al., 2007). The internal and external performance of employees in organizations are affected by LPS, which is often used in studies on role conflict, impression management, personality traits, organizational political perception, and work effectiveness (Harris et al., 2007; Brouer et al., 2011). High level of LPS effectively meets the needs and desires of subordinates, decreasing subordinates’ negative behavior, improving their work satisfaction, enhancing subordinates’ trust in leaders and loyalty to the organization, influencing the team atmosphere and promoting the relationship between employees and leaders, which in turn affects employees’ PIS (Ahearn et al., 2004; Treadway et al., 2004; Wei et al., 2010; Wang and McChamp, 2019).

Leaders are the implementer of organizational systems and often play important roles between the organization and employees. Employees often perceive that leaders’ actions are not merely individual behavior but also represent the organizations’ intentions. Leaders with a high level of political skills can effectively make employees aware of the SHRP implemented by the organization. When leaders implement management measures such as SHRP1, SHRP2, and SHRP3 for employees, making employees feel the support provided by the organization is the key for employees to form ESG and improve PIS. Leaders with satisfying political skills can make employees perceive fairness and create just organizational atmosphere. They improve employees’ loyalty and sense of belongings by making employees feel that their interests and needs are considered in the decision-making, thus affecting their PIS.

Based on the above studies, we hypothesized the moderating effect of LPS in the relationship between SHRP and PIS. To sum up, the following research hypotheses are proposed:

H5a: LPS moderates the relationship between SHRP1 and PIS, meaning that the higher the LPS, the stronger the relationship is;
H5b: LPS moderates the relationship between SHRP2 and PIS, meaning that the higher the LPS, the stronger the relationship is;
H5c: LPS moderates the relationship between SHRP3 and PIS, meaning that the higher the LPS, the stronger the relationship is.

**FIGURE 2:**
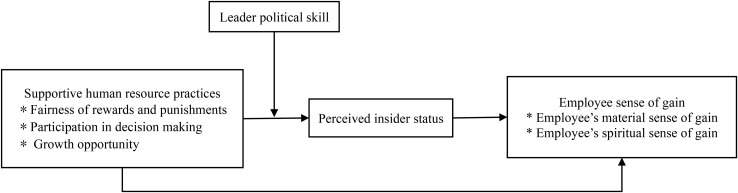
Research model.

In summary, this study reveals the influence mechanism of ESG from three aspects: organization, leadership, and individual. The conceptual model of this research is shown in Figure 2.

### Materials and Methods

#### Participants and Procedure

This study also adopted the questionnaire approach to collect data for Chinese employees. In order to reduce participants’ scruple, this study was conducted in non-working places such as non-working areas near office buildings, subway stations, and railway stations. Researchers randomly selected people in the survey site and firstly asked whether they were employees of the enterprise. Only when researchers made sure those were proper objects of the survey did researchers continue to conduct the formal survey. To ensure employees reflected their real situation, small gifts were given to the respondents. They were informed that all the questionnaires were anonymous and all information would only be used for scientific research.

A total of 287 questionnaires were issued in this survey, and 254 valid questionnaires were recovered by ruling out those which were filled in a too short period or incompletely, with an effective recovery rate of 88.502%. The basic characteristics of samples were as follows: first, gender: males (66.1%), and females (33.9%); second, age: 25 years or below (29.5%), 26–30 years (32.7%), 31–35 years old (18.9%), 36–40 years (9.4%), 41–45 years (3.9%), and 46 years or above (5.5%); third, education level: high school or below (7.9%), college graduate (37.0%), undergraduate (44.5%), and postgraduate or above (10.6%); fourth, length of service: fewer than 1 year (21.7%), 1–3 years (35.0%), 4–6 years (16.5%), 7–9 years (13.0%), 10–12 years (6.7%), and 13 years or above (7.0%); and fifth, position: senior management or high-level skills (13.0%), middle management or intermediate skill (31.5%), low-level managers or low-level skills (42.9%), and others (12.6%).

#### Measures

The ESG scale was initially developed in this study, and other variables all adopted the existing mature scales, using the Liker five-point scale, from “1” = strongly disagree to “5” = strongly agree.

##### Supportive human resource practices

Supportive human resource practices was measured with a scale used by Meyer and Smith (2000) and Allen et al. (2003). The scale was divided into three dimensions: SHRP1, SHRP2, and SHRP3. SHRP1 (Cronbach’s alpha = 0.863) was measured with a 4-item scale. A sample item was “The company implements rewards and punishments on me through performance evaluation.” SHRP2 (Cronbach’s alpha = 0.850) was measured with a 3-item scale. A sample item was “My superior will consult with me before making a decision.” SHRP3 (Cronbach’s alpha = 0.882) was measured with a 4-item scale. A sample item was “I can receive enough training.”

##### Perceived insider status

Perceived insider status (Cronbach’s alpha = 0.917) was measured with a 6-item scale, including 3 reverse-scored items, used by Stamper and Masterson (2002). The sample items were “I feel very much a part of my work organization” and “I feel like I am an ‘outsider’ at this organization (Reverse-scored).”

##### Leader political skill

Leader political skill (Cronbach’s alpha = 0.916) was measured with a 6-item scale used by Ferris et al. (1999) and Ahearn et al. (2004). It was adjusted to measure employees’ perception and feedback of their direct supervisor’s LPS based on the foundation, so as to measure the effect of LPS on employees. The sample items were “My superior finds it easy to envision himself in the position of others” and “My superior tries to find common ground with others.”

##### Employee sense of gain

ESG was measured with a 14-item scale developed by this study. The scale developed in this study was divided into two dimensions: ESG1 and ESG2. ESG1 (Cronbach’s alpha = 0.926) was measured with a 5-item scale. A sample item was “I am satisfied with the salary level in the company.” ESG2 (Cronbach’s alpha = 0.926) was measured with a 9-item scale. The sample items were “I feel satisfied psychologically working in this company” and “I can realize my self-worth by working in this company.” To further verify the ESG scale, 254 samples were tested by exploratory factor analysis and reliability analysis. After being analyzed by statistical software, the result showed that KMO = 0.928, exploratory factor analysis included two factors, and factor loadings of both factors were 0.710 or above. The total variance explained was 68.425%.

5 control variables were adopted in this study including gender, age, educational level, length of service, and position.

### Results

#### Descriptive Statistical Analysis of Variables

The descriptive statistical analysis of variables is shown in Table 3. SHRP1 was significantly positively correlated with ESG1 (*r* = 0.403, *p* < 0.001), ESG2 (*r* = 0.473, *p* < 0.001), and PIS (*r* = 0.387, *p* < 0.001). SHRP2 was significantly positively correlated with ESG1 (*r* = 0.352, *p* < 0.001), ESG2 (*r* = 0.407, *p* < 0.001), and PIS (*r* = 0.380, *p* < 0.001). SHRP3 was significantly positively correlated with ESG1 (*r* = 0.469, *p* < 0.001), ESG2 (*r* = 0.598, *p* < 0.001), and PIS (*r* = 0.473, *p* < 0.001). PIS was significantly positively correlated with ESG1 (*r* = 0.368, *p* < 0.001), and ESG2 (*r* = 0.648, *p* < 0.001).

**TABLE 3:** Means, standard deviations, and reliabilities among studied variables.

Variable	Mean	*S.D*.	1	2	3	4	5	6	7	8	9	10	11
(1) Gender	1.340	0.474	−										
(2) Age	2.421	1.389	−0.127*	−									
(3) Educational level	2.579	0.785	–0.083	0.124*	−								
(4) Length of service	2.693	1.472	−0.145*	0.626***	0.079	−							
(5) Position	2.449	0.873	−0.225***	0.489***	0.208**	0.471***	−						
(6) SHRP1	3.636	0.763	0.050	0.065	–0.071	0.048	0.108	−					
(7) SHRP2	3.120	1.003	–0.022	0.180**	0.076	0.220***	0.301***	0.467***	−				
(8) SHRP3	3.784	0.748	0.038	–0.095	–0.084	–0.071	0.122	0.585***	0.455***	−			
(9) PIS	3.973	0.670	0.023	0.067	0.040	0.145*	0.175***	0.387***	0.380***	0.473***	−		
(10) LPS	3.519	0.792	0.106	0.044	–0.036	0.046	0.028	0.439***	0.402***	0.494***	0.518***	−	
(11) ESG1	3.201	0.893	0.042	0.043	–0.040	0.106	0.090	0.403***	0.352***	0.469***	0.368***	0.410***	
(12) ESG2	3.605	0.720	0.082	–0.025	–0.073	0.056	–0.008	0.473***	0.407***	0.598***	0.648***	0.641***	0.563***

*p<0.05, **p<0.01, ***p<0.001; N = 254.

#### Confirmatory Factor Analysis

Confirmatory factor analysis was applied by applying AMOS 25.0 to assess the probability of same-source bias and examine the discriminate validity for seven variables, including SHRP1, SHRP2, SHRP3, PIS, LPS, ESG1, and ESG2. The discriminate validity of each variable was measured by comparing the quality of the fitting index measurement model. The results are shown in Table 4. It is seen from Table 4 that the fitting indexes of the seven-factor model (χ^2^ = 1,192.919, *df* = 608, χ^2^/*df* = 1.962, RMSEA = 0.062, CFI = 0.915, TLI = 0.906, SRMS = 0.0524) were significantly better than those of other models. Thus, the seven variables had good discriminative validity, indicating that they were indeed seven different constructs.

**TABLE 4:** Confirmatory factor analysis results.

Models	χ ^2^	Δχ ^2^	*df*	χ ^2^/*df*	RMSEA	CFI	TLI	SRMR
**Seven-factor model:** SHRP1, SHRP2, SHRP3, PIS, LPS, ESG1, ESG2	1,192.919	–	608	1.962	0.062	0.915	0.906	0.0529
**Six-factor model:** SHRP1 + PIS, SHRP2, SHRP3, LPS, ESG1, ESG2	1,639.596	446.677***	614	2.670	0.081	0.850	0.838	0.850
**Five-factor model:** SHRP1 + SHRP2 + PIS, SHRP3, LPS, ESG1, ESG2	1,913.950	274.354***	619	3.092	0.091	0.811	0.797	0.0845
**Four-factor model:** SHRP1 + SHRP2 + SHRP3 + PIS, LPS, ESG1, ESG2	2,181.258	267.308***	623	3.051	0.099	0.772	0.757	0.0775
**Three-factor model:** SHRP1 + SHRP2 + SHRP3 + PIS + LPS, ESG1, ESG2	2,651.531	470.273***	626	4.236	0.113	0.704	0.685	0.0873
**Two-factor model:** SHRP1 + SHRP2 + SHRP3 + PIS + LPS + ESG1, ESG2	3,292.443	640.912***	628	5.243	0.129	0.611	0.587	0.0998
**One-factor model:** SHRP1 + SHRP2 + SHRP3 + PIS + LPS + ESG1 + ESG2	3,530.457	238.014***	629	5.613	0.135	0.576	0.551	0.1036

***p < 0.001; N = 254.

#### Hypothesis Testing

In this study, the hierarchical linear regression method was used to examine the hypothesis proposed. According to the test results in Table 5, SHRP1 had a significant positive impact on ESG1 (β = 0.394, *p* < 0.001), so hypothesis H1a was supported. SHRP2 had a significant positive effect on ESG1 (β = 0.351, *p* < 0.001), so hypothesis H1b was supported. SHRP3 had a significant positive effect on ESG1 (β = 0.485, *p* < 0.00), so hypothesis H1c was supported. SHRP1 has a significant positive impact on ESG2 (β = 0.475, *p* < 0.001), so hypothesis H2a is supported. SHRP2 had a significant positive effect on ESG2 (β = 0.445, *p* < 0.001), so hypothesis H2b was supported. SHRP3 had a significant positive effect on ESG1 (β = 0.629, *p* < 0.001), so hypothesis H2c was supported. Therefore, SHRP1, SHRP2, and SHRP3 had significant positive effects on both ESG1 and ESG1. Hypotheses H1a, H1b, H1c, H2a, H2b, and H2c were all supported.

**TABLE 5:** Results of the regression analysis.

Variables	PIS				ESGI								ESG2							
			
	M1	M2	M3	M4	M5	M6	M7	M8	M9	M10	M11	M12	M13	M14	M15	M16	M17	M18	M19	M20
**Control variables**																				
Gender	0.069	0.041	0.051	0.041	0.067	0.038	0.043	0.028	0.049	0.035	0.039	0.032	0.085	0.050	0.039	0.027	0.062	0.032	0.049	0.029
Age	–0.091	–0.102	–0.086	–0.017	–0.057	–0.068	–0.024	–0.044	–0.052	–0.029	0.017	0.020	–0.089	–0.103	–0.029	–0.046	–0.083	–0.033	0.007	0.015
Educational level	0.011	0.047	0.004	0.064	–0.055	–0.018	–0.059	–0.029	–0.062	–0.063	–0.002	–0.013	–0.067	–0.022	–0.074	–0.048	–0.075	–0.078	0.002	–0.028
Length of service	0.131	0.137	0.091	0.173*	0.113	0.120	0.066	0.087	0.074	0.049	0.155*	0.127	0.125	0.133	0.038	0.056	0.075	0.022	0.180**	0.097
Position	0.171*	0.119	0.078	0.039	0.091	0.036	0.030	0.008	–0.001	–0.023	–0.042	–0.048	0.010	–0.056	–0.104	–0.123	–0.107	−0.153**	−0.163**	−0.181**
**Inde- pendent variables**																				
SHRP1		0.375***				0.394***		0.304***						0.475***		0.263***				
SHRP2			0.352***						0.351***	0.255***							0.445***	0.239***		
SHRP3				0.483***							0.485***	0.406***							0.629***	0.399***
**Mediating variable**																				
PIS							0.357***	0.240***		0.272***		0.165**			0.665***	0.564***		0.586***		0.476***
*R*^2^	0.045	0.182	0.156	0.245	0.023	0.174	0.144	0.221	0.133	0.196	0.263	0.263	0.021	0.241	0.443	0.501	0.199	0.488	0.391	0.558
*F*	2.330*	9.161***	7.623***	14.672***	1.156	8.683***	6.935***	9.990***	6.334***	8.557***	13.198***	12.529***	1.067	13.062***	32.765***	35.291***	10.228***	33.555***	26.440***	44.404***
Δ*R*^2^	0.045	0.137	0.111	0.218	0.023	0.151	0.121	0.199	0.111	0.173	0.220	0.240	0.021	0.220	0.422	0.480	0.178	0.467	0.370	0.537
Δ*F*	2.330*	41.416***	32.602***	72.999***	1.156	45.288***	35.040***	31.370***	31.515***	26.465***	71.762***	40.054***	1.067	71.519***	187.245***	118.327***	54.870***	112.379***	150.096***	149.550***

*p < 0.05, **p < 0.01, ***p < 0.001; N = 254.

This study followed the criteria by Baron and Kenny (1986) to examine the mediating effect of PIS. The mediating effect should meet the following conditions: (1) independent variables have significant influence on dependent variables; (2) independent variables have significant influence on mediating variables; (3) mediating variables have significant influence on dependent variables; and (4) when independent variables and mediating variables enter the regression equation to affect the dependent variables at the same time, the mediating variables have significant effects and the independent variables’ effects would disappear or become weakened, so that the dependent variables are completely or partially mediated by the mediating variables. The results of the stepwise regression analysis were shown in Table 5.

SHRP1 had a significant positive influence on ESG1 (β = 0.394, *p* < 0.001). SHRP1 had a significant positive influence on PIS (β = 0.375, *p* < 0.001). PIS had a significant positive influence on ESG1 (β = 0.357, *p* < 0.001). After the addition of the independent variable (SHRP1) and the mediating variable (PIS), PIS had a significant positive influence on ESG1 (β = 0.240, *p* < 0.001). Regression coefficient of the influence of SHRP1 on ESG1 reduced from β = 0.394 (*p* < 0.001) to β = 0.304 (*p* < 0.001) significantly. Thus, PIS mediated the relationship between SHRP1 and ESG1, and hypothesis H3a was supported. Similarly, SHRP2 had a significant positive influence on ESG1 (β = 0.351, *p* < 0.001). SHRP2 had a significant positive influence on PIS (β = 0.352, *p* < 0.001). After the addition of SHRP2 and PIS, PIS had a significant positive influence on ESG1 (β = 0.272, *p* < 0.001) and the regression coefficient of the influence of SHRP2 on ESG1 reduced significantly from β = 0.351 (*p* < 0.001) to β = 0.255 (*p* < 0.001). Therefore, PIS mediated the relationship between SHRP2 and ESG1, and hypothesis H3b was supported. SHRP3 had a significant positive influence on ESG1 (β = 0.485, *p* < 0.001). SHRP3 had a significant positive influence on PIS (β = 0.483, *p* < 0.001). With the entering of PIS, PIS had a significant positive influence on ESG1 (β = 0.165, *p* < 0.01). The regression coefficient of the influence of SHRP3 on ESG1 reduced significantly from β = 0.485 (*p* < 0.001) to β = 0.406 (*p* < 0.001). Therefore, PIS mediated the relationship between SHRP3 and ESG1, and hypothesis H3c was supported. As a result, hypothesis H3a, H3b, and H3c were all supported.

In the same way, SHRP1 has a significant positive influence on ESG2 (β = 0.475, *p* < 0.001). SHRP1 had a significant positive influence on PIS (β = 0.375, *p* < 0.001). PIS had a significant positive influence on ESG2 (β = 0.665, *p* < 0.001). After the addition of SHRP1 and PIS, PIS had a significant positive influence on ESG2 (β = 0.564, *p* < 0.001). The regression coefficient of the influence of SHRP1 on ESG2 reduced significantly from β = 0.475 (*p* < 0.001) to β = 0.263 (*p* < 0.001). Thus, PIS mediated the relationship between SHRP1 and ESG2, and hypothesis H4a was supported. SHRP2 had a significant positive influence on ESG2 (β = 0.445, *p* < 0.001). SHRP2 had a significant positive influence on PIS (β = 0.352, *p* < 0.001). After the addition of PIS, PIS had a significant positive influence on ESG2 (β = 0.586, *p* < 0.001), and the regression coefficient of the influence of SHRP2 on ESG2 reduced significantly from β = 0.445 (*p* < 0.001) to β = 0.239 (*p* < 0.001). Therefore, PIS mediated the relationship between SHRP2 and ESG at the spiritual level, and hypothesis H4b was supported. SHRP3 had a significant positive influence on ESG2 (β = 0.629, *p* < 0.001). SHRP3 had a significant positive influence on PIS (β = 0.483, *p* < 0.001). With the entering of PIS, PIS had a significant positive influence on ESG2 (β = 0.476, *p* < 0.001) and the regression coefficient of the influence of SHRP3 on ESG2 reduced significantly from β = 0.629 (*p* < 0.001) to β = 0.399(*p* < 0.001). Therefore, PIS mediated the relationship between SHRP3 and ESG2, and hypothesis H4c was supported. As a result, hypothesis H4a, H4b, and H4c were all supported.

In order to further verify the mediating effect of PIS, Hayes’ SPSS PROCESS macro was applied (Hayes, 2013), which specified a mediator’s model, and was consistent with the conceptual model of this article. The bootstrapping method was used to test for the mediating effect of PIS, with a sample capacity of 5000 and a 95% confidence interval for bias correction. The test results are shown in Table 6, and the confidence intervals did not include 0. Therefore, it was further verified that PIS had mediating effects between SHRP (SHRP1, SHRP2, and SHRP3) and ESG (ESG1 and ESG2). Hypotheses H3a, H3b, H3c, H4a, H4b, and H4c were supported.

**TABLE 6:** Results of the mediating effect.

Model pathways	*b*	Boot *SE*	Bootstrap 95% CI
SHRP1 → PIS → ESG1	0.105***	0.036	[0.045, 0.186]
SHRP2 → PIS → ESG1	0.085***	0.026	[0.039, 0.142]
SHRP3 → PIS → ESG1	0.095***	0.041	[0.021, 0.182]
SHRP1 → PIS → ESG2	0.200***	0.039	[0.129, 0.282]
SHRP2 → PIS → ESG2	0.148***	0.030	[0.090, 0.209]
SHRP3 → PIS → ESG2	0.221***	0.036	[0.155, 0.296]

***p < 0.001*; N = 254. Bootstrap resample = 5000. b is a non-standard regression coefficient for* indirect effect. *SE is a Std. Error. CI is a confidence interval*.

The moderating effects were tested, and results are shown in Table 7. In model 3, the interaction of SHRP1 and LPS had a significant impact on PIS (β = 0.178, *p* < 0.01). LPS moderated the relationship between SHRP1 and PIS. Therefore, hypothesis H5a was supported. In model 5, the interaction of SHRP2 and LPS had no significant impact on PIS (β = −0.009, *p* > 0.05). LPS did not moderate the relationship between SHRP2 and PIS. Hence, hypothesis H5b was not supported. In model 7, the interaction of SHRP3 and LPS had a significant impact on LPS (β = 0.114, *p* < 0.05). LPS moderated the relationship between SHRP3 and PIS. Hypothesis H5c was supported. Therefore, hypotheses H5a and H5c were supported while H5b was not.

**TABLE 7:** Regression analysis on the moderating effect of LPS.

Variables	PIS						
	
	M1	M2	M3	M4	M5	M6	M7
**Control variables**							
Gender	0.069	0.004	–0.007	0.008	0.008	0.009	0.004
Age	–0.091	–0.107	–0.080	–0.100	–0.101	–0.056	–0.044
Educational level	0.011	0.045	0.065	0.025	0.025	0.057	0.075
Length of service	0.131	0.119	0.084	0.098	0.098	0.144	0.128
Position	0.171*	0.131*	0.133	0.114	0.116	0.079	0.075
**Independent variables**							
SHRP1		0.188**	0.210***				
SHRP2				0.159*	0.159*		
SHRP3						0.291***	0.316***
**Moderating variable**							
LPS		0.432***	0.454***	0.451***	0.449***	0.369***	0.386***
**Interactions**							
SHRP1* LPS			0.178**				
SHRP2* LPS					–0.009		
SHRP3* LPS							0.114*
*R*^2^	0.045	0.331	0.360	0.322	0.322	0.362	0.373
*F*	2.330*	17.402***	17.251***	16.704***	14.562***	19.932***	18.235***
Δ*R*^2^	0.045	0.286	0.029	0.277	0.000	0.317	0.011
(△F	2.330*	52.658***	11.160**	50.323***	0.028	61.114***	4.418*

*p < 0.05, **p < 0.01, ***p < 0.001; N = 254.

**FIGURE 3:**
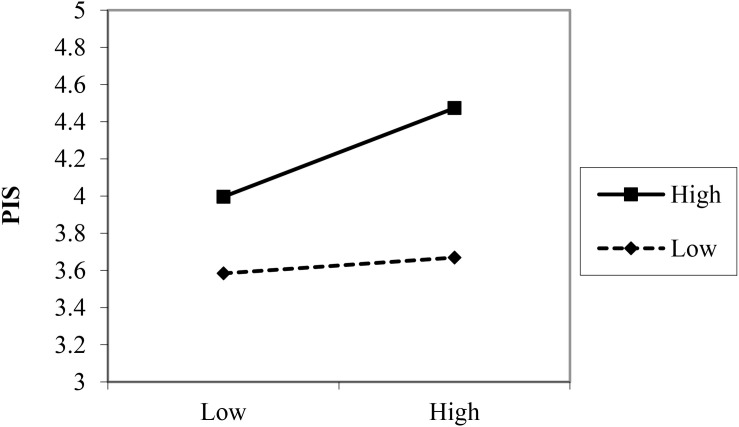
The moderating effect of LPS on the relationship between SHRP1 and PIS.

**FIGURE 4:**
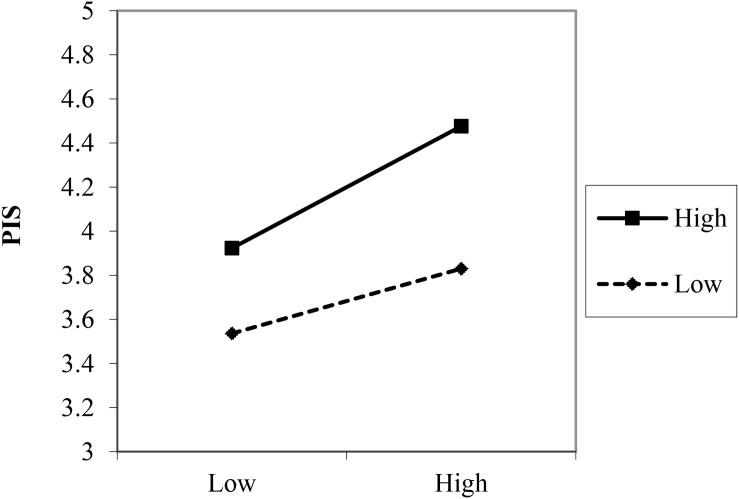
The moderating effect of LPS on the relationship between SHRP3 and PIS.

**FIGURE 5:**
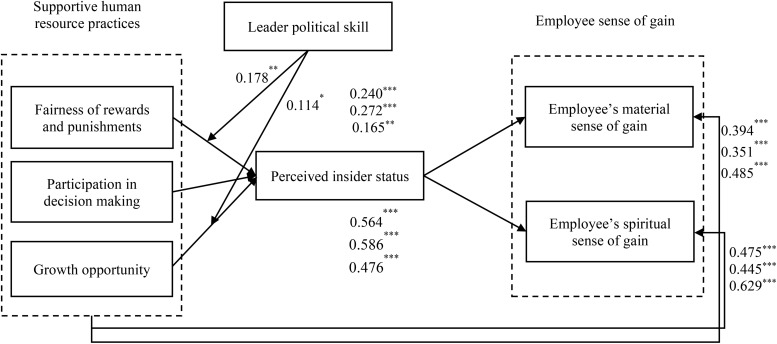
Test results. *p < 0.05, **p < 0.01, ***p < 0.001; *N* = 254.

To further analyze how the interactions between LPS and SHRP affected PIS, Hayes’ SPSS PROCESS macro was applied (Hayes, 2013). In addition, the moderating effect charts of LPS were added, to clarify the moderating effect of LPS (see Figures 3, 4). Under the test of Process (*M* ± *1SD*). The positive relation between SHRP1 and PIS was not significant (β = 0.056, *SE* = 0.060, *p* = 0.376) when the level of LPS was low (*M − 1SD*), while it became significant (β = 0.312, *SE* = 0.067, *p* < 0.001), when the level of LPS was high (*M* + *1SD*), indicating that the higher the LPS, the stronger the relationship was. Thus, the positive moderating effect of LPS on the relationship between SHRP1 and PIS is shown in Figure 3. Hypothesis H5a was further supported. Under the test of Process (*M* ± *1SD*), 95% confidence interval (*CI* = [−0.094, 0.079]) of the interaction of LPS and SHRP2 included 0, *p* > 0.05. Thereby, LPS had no moderating effect on the relationship between SHRP2 and PIS. Hypothesis H5b was not supported. The positive relation between SHRP3 and PIS was weak (β = 0.197, *SE* = 0.062, *p* < 0.001) when the level of LPS was low (*M − 1SD*), while it became strong (β = 0.370, *SE* = 0.075, *p* < 0.001), when the level of LPS was high (*M* + *1SD*), indicating that the higher the LPS, the stronger the relationship was. Thus, the positive moderating effect of LPS on the relationship between SHRP3 and PIS is shown in Figure 4. Hypothesis H5c was further supported. To sum up, hypotheses H5a and H5c were further supported yet H5b was not verified. This study assumed that the test results were summarized as shown in Figure 5.

### Study 2 Discussion

Study 2 investigated the antecedents and mechanism of ESG based on statistical analysis of 254 valid questionnaires from the perspective of SHRP. Through questionnaires and empirical research, it was found that all dimensions of SHRP, including SHRP1, SHRP2, and SHRP3, had significant positive effects on dimensions of ESG, consisting of ESG1 and ESG2. PIS played a partial mediating role between all dimensions of SHRP and ESG. LPS positively affected the relationship between SHRP1 and PIS, as well as the relationship between SHRP3 and PIS.

## General Discussion

Although practitioners have begun to embrace the notion of ESG in public governance efforts, research still lags behind in understanding the concept’s nature and role for the human resource management. Two studies explored the concept of ESG and a scale measuring ESG and investigated the antecedents and mechanism of ESG in the Chinese setting workplace. Study 1 developed an integrative framework of concept of ESG and reported the development and validation of a scale measuring ESG, based on the multiphase scale development process results in a 14-item ESG scale measuring two dimensions: ESG1 and ESG2. Study 2 investigated the antecedents and influence mechanism of ESG, as well as boundary conditions. Study 2 showed that ESG was influenced by the all three dimensions of SHRP, whereby the influences were mediated by PIS. Results also suggested that LPS moderated the effect between SHRP (SHRP1 and SHRP3) and PIS positively.

## Theoretical Implications

First of all, this article reviewed research related to sense of gain and applied it to the organizational context. The concept of ESG was defined, and the scale was developed accordingly. President Xi Jinping of China put forward the policy that required the full demonstration on providing all people more sense of gain. Thus, improving people’s sense of gain has become an important topic in China in recent years (Cao and Li, 2017; Zheng and Chen, 2017). This article enriches the academic study on sense of gain and helps to promote the theoretical research on ESG. ESG is defined as the subjective feeling of getting various objective benefits due to employees’ efforts at work.

Based on the indicators for sense of gain proposed by “Chinese economic life survey (2017–2018), the Better Life Index,” a scale for sense of gain in the organizational context was developed through expert discussion, exploratory factor analysis, reliability analysis, and confirmatory factor analysis. The scale is composed of two dimensions and 14 items. ESG1 includes 5 items referring to the sense of material gain in terms of material factors such as salary and benefit level, or old age, housing, and medical security. ESG2 includes 9 items referring to the sense of gain caused by psychological factors including organizational culture, the relationships between colleagues, leisure-time life, self-worth, and power of example.

Finally, this article explored the influence mechanism of ESG from the perspective of SHRP and provided theoretical evidence for ESG. Firstly, it is found in the study that all dimensions of SHRP (SHRP1, SHRP2, and SHRP3) had significant positive effects on all dimensions of ESG (ESG1 and ESG2). Secondly, PIS played a partial mediating role between all dimensions of SHRP and ESG. Through SHRPs such as SHRP1, SHRP2, and SHRP3, the organization shows that it recognizes, supports, and respects employees (Allen et al., 2003). When employees perceive organizational support, their PIS will be stimulated (Stamper and Masterson, 2002). Therefore, they are willing to contribute more to the organization (Rhoades and Eisenberger, 2002; Chen and Aryee, 2007), so as to enhance their ESG. Thirdly, LPS positively moderated the relationship between SHRP1 and PIS, as well as the relationship between SHRP3 and PIS. The supportive measures provided by organizations are affected by LPS (Ahearn et al., 2004). Leaders with a high level of LPS can put themselves into employees’ shoes, increasing the positive effect of organizational support, and enhancing employees’ PIS.

## Managerial Implications

This article explored the influencing mechanism of ESG from three perspectives: organization (SHRP), leadership (LPS), and individual (PIS). This provides the theoretical bases and implications for management practice of enhancing ESG.

First is creating a fair atmosphere of rewards and punishments. This study conducted that SHRP1 can help improve ESG. The salary level and welfare level are important indicators of ESG. Rewards and punishments involve the immediate interests of employees. The organization should strictly formulate scientific and reasonable performance appraisal systems, rewards and punishment systems, and other systems related to the immediate interests of employees. Also, the implementation shall be carried out by managers in accordance with the unified standard without exceptions. Employees perceive the organization’s fairness in the whole management process and the practice of rewards and punishments system, thus strengthening ESG (Shi, 2017).

Second is constructing an employee decision-making participation mechanism. Establishing the employee decision-making participation mechanism improves the internal information communication channels and enhances the interaction between the organization and employees. When major decisions are made, the organization can let employees engage properly, consider their feelings, and promote their sense of participation and belonging. Establishing and improving information communication channels to build a platform for employees to share their opinions, ideas, and suggestions can increase the interactions and understanding between organizations and employees.

Third is promoting career development of employees. Promoting the career development of employees and the growth of employees themselves increases the actual gain of employees. Every employee pays attention to the career development. The organization should also provide adequate and specific training for employees in different positions to improve the necessary skills required by certain positions and at the same time help them continuously learn new knowledge and technologies. As the superior, leaders should communicate with their subordinates, provide guidance on their difficulties at work, and improve their working skills and work efficiency. Employees are likely to achieve professional progress and career development, realize their self-worth, and enhance their sense of gain.

Fourth is developing the LPS of leadership. People from the management level who have the most frequent and direct contact with employees are their direct leaders. Leaders with high LPS can effectively influence and motive their subordinates (Ahearn et al., 2004). Leaders often play great roles in providing support when employees are aware of the organization’s behavior (Eisenberger et al., 1986). As one of the competencies that managers should have, the organization should take targeted measures such as training to improve the political skill of leaders. At the same time, the organization should take the political skill into evaluation and promotion indicators of leaders. Also, leaders should set examples and motivate their employees to make progress. Leaders play an exemplary role in the organization, with their strong personal charm, charisma, and appeal. Employees are often willing to follow such leaders and strive with them. Employees who perceive the support given by the organization will be treated as part of the organization, and they would make more efforts and thus get more gains (Chen and Aryee, 2007). Lastly, leaders should enhance their sense of service and efficiency toward employees to cultivate their sense of belonging. Based on the organizational support theory, employees will try their best to repay the leaders who have helped them. If leaders are good at communicating, employees are often more willing to interact with them. If employees approve leaders to some degree, they will approve their organization to a certain extent and try their best to work and repay it.

Finally is enhancing employees’ PIS and organizational identification. Leaders are the implementers of organizational measures and directly affect the perception of employees’ insider status. When leaders show supports and concerns toward employees would lead to employees perceive the approval and attention of superiors. It can make employees consider themselves as a part of the organization and increase their PIS and recognition for the organization (Wang et al., 2006). In this way, their ESG can be promoted.

## Limitations and Future Suggestions

The limitations of this study include the following three aspects. Firstly, due to limited time, this study directly used the target concerning the sense of gain in “Chinese economic life survey (2017–2018), the Better Life Index” to convert the organizational level indicators in this study. The deductive method to develop the scale of ESG was used without acquiring the primary data about ESG. Future research might apply the grounded theory and other methods to improve the scale after obtaining primary data from the case enterprises. Secondly, due to the limited funds, the survey samples of the study were limited to one city in a country. Follow-up studies can select survey samples more widely in different cities, countries, or different cultural and economic backgrounds to verify and improve the results. Thirdly, the mechanism of antecedents to explore ESG was confined to a single level instead of analyzing it in more views. In the follow-up studies, cross-layer analysis can be conducted such as the organizational level and leader’s level.

## Conclusion

This article introduces sense of gain, a political term into organizational context, and systematically discusses various important questions such as its definition, its measurement, and its way of generating. Firstly, this study defined ESG as the subjective feeling of getting various objective benefits due to employees’ efforts at work. This definition is embedded with three implications: efforts at work serve as the precondition for ESG; various objective benefits are the basis of ESG; and subjective feelings are viewed as the kernel of ESG. Next, based on the indicators for sense of gain proposed by “Chinese economic life survey (2017–2018), the Better Life Index,” a scale with 14 items for sense of gain in organizational context was developed. Finally, this study explored the influence mechanism of ESG. In this study, it is verified that SHRP including SHRP1, SHRP2, and SHRP3 had significant positive effects on dimensions of ESG consisting of ESG1 and ESG2. PIS played a partial mediating role between the relationships mentioned above. LPS moderated the relationship between SHRP1 and PIS, as well as the relationship between SHRP3 and PIS. Lastly, this study proposed management measures to enhance ESG from the perspectives of organizations, leaders, and employees.

## Data Availability Statement

The raw data supporting the conclusions of this article will be made available by the authors, without undue reservation, to any qualified researcher.

## Ethics Statement

Ethical review and approval was not required for the study on human participants in accordance with the local legislation and institutional requirements. The patients/participants provided their written informed consent to participate in this study.

## Author Contributions

YG and YY: conceptualization, methodology, and writing – original draft. YY and JW: data curation and formal analysis. YG, YY, and JW: investigation and writing – review and editing. YY: project administration. YG: supervision. All authors contributed to the article and approved the submitted version.

## Conflict of Interest

The authors declare that the research was conducted in the absence of any commercial or financial relationships that could be construed as a potential conflict of interest.
